# Prevalence and determinants of inconsistent condom use among unmarried sexually active youth. a secondary analysis of the 2016-2017 Eswatini HIV incidence measurement survey

**DOI:** 10.4314/ahs.v23i1.42

**Published:** 2023-03

**Authors:** Maswati S Simelane, Garikayi B Chemhaka, Fortunate S Shabalala, Portia T Simelane, Zodwa Vilakati

**Affiliations:** 1 Department of Statistics and Demography, Faculty of Social Sciences, University of Eswatini, Kwaluseni, Eswatini; 2 Department of Community Health Nursing Science, Faculty of Health Sciences, University of Eswatini, Mbabane, Eswatini; 3 Department of Human and Social Sciences, Townsville, Education Office, Queensland, Australia; 4 Department of Population Studies, University of Zambia

**Keywords:** Inconsistent condom use, youth, factors, Eswatini

## Abstract

**Introduction:**

In sub-Saharan Africa, youth continue to have a high burden of sexually transmitted infections and unplanned pregnancies that could be reduced through improved condom use. We aimed to investigate inconsistent condom use with the three most recent sexual partners among unmarried sexually active youth in Eswatini

**Methods:**

Data were analysed for 1,324 youth from the 2016-2017 Swaziland HIV incidence measurement survey (SHIMS2) using multivariable logistic regression method.

**Results:**

The prevalence of inconsistent condom use among youth was 56.8% in 2017. Higher odds of inconsistent condom use were observed among youth aged 20-24, residents in the Lubombo region, those aged less than 18 years at sexual debut and those who had two or more sexual partners in the last 12 months before the survey. Lower odds of inconsistent condom use were observed among males, and those that knew their partners reported HIV status to be negative.

**Conclusion:**

Our findings highlight a notable prevalence of inconsistent condom use among youth in Eswatini. Programs that aim to reduce the incidence of HIV infections and STIs among youth in Eswatini should focus on these factors associated with inconsistent condom use.

## Introduction

Human immunodeficiency virus (HIV) is a global public health problem that disproportionately affects young people in resource-limited settings. In sub-Saharan Africa (SSA), HIV remains the main cause of morbidity and mortality^1^. The global community has established several policies to combat the spread of HIV and AIDS-related mortality primarily in SSA where about 75% of all deaths, two-thirds of all new infections occur, and had 71% of people living with HIV in 2017^1^,^2^. Post-2015, the sustainable development goals (SDGs) especially number 3 aim to ensure healthy lives and promote well-being for all across all ages and end the epidemic by 2030^3^.

Regardless of the observed decline in the HIV incidence across all ages in SSA, gaps still exist across sex and age groups^4^. Among the people living with HIV/AIDS, youth are at the center of the epidemic^5^,^6^. It is evident that in SSA, youth continue to have a high burden of HIV, and sexually transmitted infections (STIs) that could be reduced through improved strategies such as consistent condom use^7^, ^8^. When used consistently and correctly, the male condom is effective in reducing sexual transmission of HIV and STIs 9. As such, countries that reported effective condom use have consistently reported lower HIV prevalence relative to those that reported lower use of condoms^10^,^11^.

Unprotected sexual intercourse is an important factor that explains the high HIV infection, STIs and unplanned pregnancies especially among unmarried young adults^12^–^14^. This sub population is more vulnerable to having multiple sexual partners when compared to the married^15^. Engaging in sexual intercourse other than a spouse or cohabiting is considered to be high risk sex that predisposes individuals to STIs and unwanted pregnancy^15^. Youth practice sexual risk behaviours such as multiple sexual partners in order to increase their latitude in choosing who to marry as they mature^16^. Such sexual networks increase the risk of HIV transmission among sexually active unmarried youth primarily among those in SSA^16^, where a quarter of the adolescents engage in first sex before the age of 15 years, although there is variation across countries^17^,^18^. Evidence has shown that more than 50% of the new HIV infections reported in SSA are among the youth^17^, ^19^ with young females disproportionately affected by HIV/AIDS due to cultural, economic, social, and structural factors that predispose them to unprotected sexual intercourse^14^.

Eswatini has the highest HIV prevalence (27.3%) in the world among people aged 15-49 years^20^, ^21^. Even though Eswatini reached the United Nations 95-95-95 target in 2020, there is a need to sustain the remarkable achievement by reducing new infections by 17% among youth 22. A population-based survey conducted in Eswatini showed that in 2017 the HIV prevalence among the youth was 9.1%, three times higher among females (13.9%) compared to males (4.1%) and the prevalence was higher among those aged 20-24 years (4.2% males, 20.9% females) than those aged 15-19 years (3.9% males, 7.2% females)^20^.

The high HIV acquisition among youth could be attributed to the non-use of condoms at sexual debut which is also associated with subsequent sexual trajectories^23^. Several studies on condom use among adolescents have shown that early condom use is associated with condom use in subsequent sexual encounters 24, 25. Several factors such as age, sex, education level, occupation, household wealth index, and age at first sex are associated with condom use among youth 26, 27. It is therefore important to investigate the factors associated with inconsistent condom use to inform programs targeted to the youth. This study aimed to determine the factors associated with inconsistent condom use among youth in Eswatini.

## Data source and study design

For this analysis, we used the 2016-2017 Swaziland HIV incidence measurement survey2 (SHIMS2) data. The SHIMS2 was a community-based, nationally representative data collected from August 30, 2016, to March 31, 2017. SHIMS applied a two-stage stratified cluster sampling technique. The first stage involved the selection of 286 enumeration areas (EAs) from a sampling frame of 2064 EAs across the four administrative regions of Eswatini, which are Hhohho, Manzini, Shiselweni, and Lubombo. Second, an average sample of 20 households was selected using the probability proportional to size (PPS). A total of 11,673 adults aged 15 to 49 years old were eligible respondents and were included in the survey. Of these 3,797 were aged 15 to 24 years, 1,912 had a history of sexual intercourse in their lifetime. However, only 1324 of these were included in the analysis because they had complete data in all other characteristics used in the study.

## Study variables

### Outcome variable

The outcome variable for this study was inconsistent condom use in the last 12 months before the survey. The youth were asked about the last three (3) recent persons they had sex with: In the last 12 months, how often did you use condoms when having vaginal sex? Was it always, most of the time, sometimes, rarely, or never? It was generated to a binary variable coded as “1” when the youth reported most of the time, sometimes and rarely or never, and “0” when they reported that they used it always.

### Explanatory variables

The explanatory variables were selected based on the literature 28, 29. The socio-demographic variables were age (15-19, 20-24), sex (male or female), highest education level (no education, primary, secondary, high, tertiary). In Eswatini the education system is classified into five categories: no education (no formal education), primary (grade 1-7), secondary (form 1-3, equivalent to grade 8-10), high (form 4-5, equivalent to grade 11-12), tertiary (post high school education such as colleges, universities, technical training institutes, vocational schools). Working status in the past 12 months before the survey (employed, not employed), and household wealth index classified as poorest/ poor, middle, rich/ richest. The household wealth index was already calculated in the SHIMS2 dataset as quantiles^20^. The household wealth index is derived from durable assets such as housing materials, toilet or latrine access, phone ownership, or agricultural land and livestock, which are regularly collected in most household surveys to create an index of household wealth 30. The principal component analysis (PCA) was used to develop the household wealth index 30. The place of residence was categorized as rural and urban, and the region of residence was classified as Hhohho, Manzini, Shiselweni, and Lubombo. The behaviour-related variables were age at first sexual intercourse (less than 18 and 18 years and older), number of sexual partners last 12 months (one, two and more) and known reported partners' HIV status classified as positive, negative and never tested.

### Statistical analysis

Statistical analyses were performed using Stata version 15, Stata Corp LP, Texas. Sample distribution was assessed using frequencies and percentages. Bivariable analysis was performed using the Pearson Chi-square to test the independence of the distribution between the independent variables and inconsistent condom use. A multi-collinearity test was done, and there were no variables with a variance inflation factor (VIF) of greater than 10. A bivariable logistic regression was used to assess the crude relationship between each explanatory variable and inconsistent condom use. Only variables that were significant at p<0.20 were entered into the multivariable logistic regression model regardless of whether they were significant or not in the bivariable models. In the final model, results were reported using adjusted Odds Ratios (AORs) and 95% Confidence Intervals (95% CIs). All analyses performed in this study were weighted for probability sampling and non-response, to account for multi-stage sampling and stratification in the SHIMS2.

### Ethical Considerations

The SHIMS team granted permission for the access and use of the SHIMS dataset from https://phia-data.icap.columbia.edu/login. The dataset was anonymous and do not allow the identification of participants. The data is publicly available and the authors had no special access privileges to the data and that other researchers will be able to access the data in the same manner as the authors.

## Results

Of the total sample of this study, a majority (91.6%) were aged 20-24 years with high school education (41.8%) and 65.5% were not employed. About four in ten (43.5%) of the youth were from poor/poorest households and a majority (72.5%) were residents in rural areas. Slightly above half (54.2%) of the youth had their first sex when they were aged 18 years and older and a majority (77.7%) knew their partners' reported HIV status to be negative. The youth were almost equally distributed by sex.

The prevalence of inconsistent condom use among youth was 56.8%. A majority (94.1%) of youth who used the condom inconsistently were aged 20-24 years vs only 5.9% among those aged 15-19 years (p=0.001). The practice of inconsistent condom use was significantly different between males (43.2%), vs females (56.8%), p<0.002. There was a significantly higher proportion of youth that used condoms inconsistently among those that were not employed (62.9%) vs. 37.1% among the employed, p=0.037. About four in ten of the youth who used a condom inconsistently were from the Manzini vs. 15.1% in the Lubombo region, p<0.010. A majority (73.1%) of the youth that used a condom inconsistently had one sexual partner compared to slightly above a quarter (26.9%) who reported having two or more partners, p=0.001. Approximately, eight in ten of those who used a condom inconsistently knew their partners' HIV status to be negative while only one in ten of their partners reported to have not been tested for HIV, p<0.001. (Table 1).

**Table 1:** Sample distribution and prevalence of inconsistent condom use by the several factors

	Sample distribution	Inconsistent condom use		Chi-square
Characteristics	n (%)	Yes (%)	No (%)	p-value
**Total**	1324			
**Prevalence**		733 (56.8)	591 (43.2)	
**Age**				0.001
15–19	129 (8.4)	50 (5.9)	79 (11.7)	
20–24	1195 (91.6)	683 (94.1)	512 (88.3)	
**Sex**				0.002
Male	571 (47.6)	276 (43.2)	295 (53.4)	
Female	753 (52.4)	457 (56.8)	296 (46.6)	
**Highest Education Level**				0.477
No education	12.0 (0.9)	8 (1.0)	4 (7.0)	
Primary	218 (15.5)	120 (15.5)	98 (15.5)	
secondary	448 (32.1)	262 (33.9)	186 (29.8)	
High	536 (41.8)	283 (40.2)	253 (43.9)	
Tertiary	110 (9.7)	60 (9.4)	50 (10.0)	
**Employment status in the past 12** **months**				0.037
Employed	437 (34.5)	258 (37.1)	179 (31.1)	
Not employed	887 (65.5)	475 (62.9)	412 (68.9)	
**Household wealth index**				0.581
Poor/Poorest	611 (43.5)	349 (44.7)	262 (41.9)	
Middle	313 (23.7)	166 (23.2)	147 (24.4)	
Rich/Richest	400 (32.8)	218 (32.1)	182 (33.7)	
**Place of residence**				0.066
Rural	1027 (72.5)	174 (29.8)	123 (24.5)	
Urban	297 (27.5)	559 (70.2)	468 (75.5)	
**Region of residence**				0.010
Hhohho	402 (29.6)	203 (26.8)	199 (33.3)	
Manzini	254 (18.1)	158 (20.0)	96 (15.5)	
Shiselweni	245 (16.9)	251 (38.1)	172 (31.9)	
Lubombo	423 (35.4)	121 (15.1)	124 (19.3)	
**Age at first sex (in years)**				0.032
Less than 18	632 (45.8)	365 (48.5)	267 (42.3)	
18 and older	692 (54.2)	368 (51.5)	324 (57.7)	
**Number of sexual partners last 12** **months**				0.001
One	1050 (77.7)	549 (73.1)	501 (83.7)	
two and above	274 (22.3)	184 (26.9)	90 (16.3)	
**Known reported partners HIV** **status**				0.001
Positive	113 (7.7)	61 (7.6)	52 (7.8)	
Negative	1029 (77.7)	597 (81.2)	432 (73.0)	
Never tested	182 (14.6)	75 (11.1)	107 (19.2)	

### Determinants of inconsistent condom use

Table 2 shows the bivariable and multivariable findings for inconsistent condom use among the youth. In the bivariable model, age, sex, employment status in the past 12 months, region of residence, and known reported partners’ HIV status were significantly associated with inconsistent condom use among youth. After controlling for other factors in the multivariable model, age, sex, region of residence, age at first sex, number of sexual partners in the last 12 months before the survey, and known reported partners' HIV status were associated with inconsistent condom use. Higher odds of inconsistent condom use were observed among youth aged 20-24, (AOR=2.84, 95% CI: 1.81, 4.44) compared to those aged 15-19 years. Lower odds of inconsistent condom use were observed among males (AOR=0.54, 95% CI: 0.42, 0.70) compared to females. The youth residing in the Lubombo region had higher odds of inconsistent condom use (AOR=1.60, 95% CI: 1.04, 2.45) compared to those residing in the Hhohho region. Even after controlling for other factors in the model, higher odds of inconsistent condom use were observed among youths that were aged less than 18 years at sexual debut (AOR=1.57, 95% CI:1.22,2.01) compared to those that were aged 18 years and older at sexual debut. Youth that had two or more sexual partners in the last 12 months before the survey had higher odds of practicing inconsistent condom use (AOR=2.21, 95% CI: 1.54, 3.16) compared to those that had one sexual partner. Lower odds of inconsistent condom use were observed among youth that knew their partners reported HIV status to be positive (AOR=0.63, 95% CI: 0.42, 0.96) and those that never tested for HIV (AOR=0.61, 95% CI: 0.44, 0.85) compared to those that knew their partners reported HIV status to be negative.

**Table 2:** Factors associated with inconsistent condom use among unmarried young people

Characteristics	OR (95%CI)	p-value	AOR (95%CI)	p-value
**Age**				
15–19	1		1	
20–24	2.11 (1.41,3.16)	0.001	2.84 (1.81,4.44)	<0.001
**Sex**				
Male	0.66 (0.52,0.85)	0.002	0.54 (0.42,0.70)	<0.001
Female	1		1	
**Highest Education Level**				
No education	1.47 (0.43,5.09)	0.525	1.74 (0.40,7.54)	0.447
Primary	1.07 (0.70,1.62)	0.755	1.37 (0.83,2.27)	0.206
secondary	1.21 (0.82,1.78)	0.314	1.43 (0.92,2.22)	0.103
High school	0.98 (0.82,1.78)	0.889	1.12 (0.74,1.69)	0.582
Tertiary	1		1	
**Employment status in the past 12** **months**				
Employed	1		1	
Not employed	0.76 (0.59,0.98)	0.038	0.80 (0.61,1.05)	0.106
**Household wealth index**				
Poor/Poorest	1.12 (0.86,1.45)	0.378	-	
Middle	1.00 (0.74,1.35)	0.999	-	
Rich /Richest	1			
**Place of residence**				
Rural	0.77 (0.58,1.02)	0.068	0.74 (0.52,1.04)	0.080
Urban	1		1	
**Region of residence**				
Hhohho	1		1	
Manzini	1.48 (1.05,2.40)	0.023	1.42 (1.00,2.04)	0.053
Shiselweni	0.97 (0.68,1.39)	0.024	0.93 (0.64,1.26)	0.702
Lubombo	1.60 (1.07,2.08)	0.874	1.60 (1.04,2.45)	0.032
**Age at first sex (in years)**				
Less than 18	1.29 (1.02,1.61)	0.032	1.57 (1.22,2.01)	0.001
18 and older	1		1	
**Number of sexual partners last 12** **months**				
One	1		1	
Two and above	1.89 (1.35,2.65)	0.001	2.21 (1.54,3.16)	<0.001
**Known reported partners HIV status**				
Positive	0.88 (0.59,1.32)	0.523	0.63 (0.42,0.96)	0.034
Negative	1		1	
Never tested	0.52 (0.39,0.70)	<0.001	0.61 (0.44,0.85)	0.004

Notes: OR=crude odds ratios; AOR=adjusted odds ratios; p<0.05; *Multivariable logistic model fit statistic (χ^2^)* =0.5851

## Discussion

This study reported slightly above half of the youth used condoms inconsistently in Eswatini and several factors were associated with the inconsistent condom use. The magnitude of inconsistent condom use in Eswatini is similar to that reported in Zambia (59%) 31 and Nigeria (61.4) 14, but slightly higher than that reported in South Africa (48%) 11, 32. The higher prevalence of inconsistent condom use in developing countries including Eswatini may be due to the relaxation of HIV and AIDS preventive practices targeting youth. For example, in Cameroon, more efforts and funding were directed to initiating patients on antiretroviral treatment (ART) than preventive programs such as condom use^33^. Therefore, the importance of continued interventions that aim to reduce sexually transmitted infections, such as HIV through consistent condom use among people engaging in risky sexual behaviours cannot be over-emphasized.

Similar to a study conducted in South Africa among same-sex partners^34^, we found that youth aged 20-24 years had higher odds of practicing inconsistent condom use compared to those aged 15-19 years. Our findings may point out more effective HIV interventions for adolescents who are attending school^35^ while youth aged 20-24 years may have limited access to prevention and care services.

This study showed significant sex differences in condom use behaviour among the youth. For example, young males had lower odds of practicing inconsistent condom use compared to their female counterparts. This study's findings are similar to other studies^36^, ^37^. This could be due to that, young women are more likely to be involved in intergenerational sex, where they engage in sexual intercourse with older men^36^, ^38^, ^39^. Young women may have less power to negotiate for safer sex with older men^37^. This in turn leads to high unwanted pregnancy and sexually transmitted infections.

Youth from the Lubombo region had higher odds of practicing inconsistent condom use when compared to the Hhohho region. A possible explanation could be that the Lubombo region is the poorest in Eswatini, with a poverty rate of 71.5% vs 54.1% in the Hhohho region in 2017^40^. It has long been established that poverty increases sexual risky behaviours including inconsistent condom use which in turn predisposes people to HIV^41^, ^42^.

Engaging in sexual activity at younger ages results in higher odds of various harmful outcomes such as inconsistent condom use^43^, ^44^. Similar to other studies^45^–^47^, we found that youth that engaged in sexual intercourse when they were aged less than 18 years old had higher odds of inconsistent condom use.

Our study showed that youth who had two or more sexual partners had higher odds of inconsistent condom use when compared to those that had only one sexual partner. The findings collaborate findings done in Nigeria^14^. Other studies found that people with multiple sexual partners use condoms more frequently than people with a single partner^48^, ^49^. The practice of inconsistent condom use among youth in Eswatini poses a health risk considering that a significant amount of the youth in the past 12 months before the survey had multiple sexual partners (see table 1), indicating a need for public health programs. Inconsistent condom use especially with multiple sexual partners is a risk factor for STIs and unplanned and unwanted pregnancies. Our study findings have broad implications for the sexual and reproductive health of the youth in Eswatini.

This study established that youth who knew their sexual partners to be HIV positive were less likely to practice unsafe sexual intercourse. Our findings are in line with other studies elsewhere that found that young people who knew their partners to be HIV positive were less likely to practice inconsistent condom use^14^. This suggests that encouraging couple testing and conversation on HIV and STIs among youth could be an effective strategy for increasing the practice on consistent condom use^50^.

## Strengths and weaknesses

The study findings have a number of limitations. The outcome variable was self-reported, which may be prone to social desirability and recall bias. The temporal changes in inconsistent condom use and the causality could not be demonstrated with cross-sectional data. The use of the household wealth index to estimate the socioeconomic status could be critiqued; however, developing countries similar to Eswatini lack valid data on income and expenditure. Moreover, the explanatory variable, known partner's HIV status was self-reported which could have introduced social desirability bias or participants could have used their general knowledge to classify their partners' HIV status. However, the study provided important findings on the prevalence of inconsistent condom use among the youth including the associated factors. This will allow policymakers and researchers to evaluate the effectiveness of existing HIV interventions such as the availability and use of condoms among the youth.

## Conclusion

In this study, we found just slightly above half of youth who used a condom inconsistently and that age, sex, region of residence, age at first sex, number of sexual partners last 12 months before the survey and known reported partners' HIV status were significantly associated with inconsistent condom use. The findings imply that consistent condom use could be improved if interventions consider the factors found to be associated with inconsistent condom use.

## Data Availability

The population-based HIV impact assessment (PHIA) team granted access and use of the SHIMS2 dataset from https://phia- data.icap.columbia.edu/files.
